# Physiological Stimuli Induce PAD4-Dependent, ROS-Independent NETosis, With Early and Late Events Controlled by Discrete Signaling Pathways

**DOI:** 10.3389/fimmu.2018.02036

**Published:** 2018-09-18

**Authors:** Olga Tatsiy, Patrick P. McDonald

**Affiliations:** Pulmonary Division, Faculty of Medicine, Centre de recherche du CHUS and Université de Sherbrooke, Sherbrooke, QC, Canada

**Keywords:** neutrophils, extracellular traps, signaling, NADPH oxidase, protein arginine deiminase

## Abstract

Neutrophils are known to extrude decondensed chromatin, thus forming NETs (neutrophil extracellular traps). These structures immobilize pathogens, thereby preventing their spreading, and are also adorned with antimicrobial molecules. NETs can also influence pathogenesis in chronic inflammation, autoimmunity, and cancer. Despite the importance of NETs, the molecular mechanisms underlying their formation, as well as the upstream signaling pathways involved, are only partially understood. Likewise, current methodological approaches to quantify NETs suffer from significant drawbacks, not the least being the inclusion of a significant non-specific signal. In this study, we used novel, fluorescent polymers that only bind extruded chromatin, allowing a specific and standardized quantification of NETosis. This allowed us to reliably rank the relative potency of various physiologic NET inducers. In neutrophils activated with such stimuli, inhibition of the Syk or PI3K pathways blocked NETosis by acting upon late events in NET formation. Inhibition of the TAK1, p38 MAPK, or MEK pathways also hindered NETosis, but by acting on early events. By contrast, inhibiting PKC, Src family kinases, or JNK failed to prevent NETosis; cycloheximide or actinomycin D were also ineffective. Expectedly, NET formation was deeply compromised following inhibition of the NADPH oxidase in PMA-activated neutrophils, but was found to be ROS-independent in response to physiological agonists. Conversely, we show for the first time in human neutrophils that selective inhibition of PAD4 potently prevents NETosis by all stimuli tested. Our data substantially extends current knowledge of the signaling pathways controlling NETosis, and reveals how they affect early or late stages of the phenomenon. In view of the involvement of NETs in several pathologies, our findings also identify molecular targets that could be exploited for therapeutic intervention.

## Introduction

Neutrophils are a cornerstone of the innate immune system, by virtue of their phagocytic and microbicidal activities, which greatly contribute to pathogen clearance. In this context, an important neutrophil response is their ability to extrude decondensed chromatin, thus forming extracellular structures termed NETs (for neutrophil extracellular traps) (1). The chromatin backbone of NETs entraps various microorganisms (bacteria, viruses, yeasts, and even some parasites) (1–3), and while DNA itself can exert antimicrobial effects (4), NETs feature histones, proteases, and other components, which all participate in microorganism killing. The ability of neutrophils to undergo NETosis is conserved across vertebrates, from zebrafish to mammals, and has been observed in several *in vivo* settings, suggesting that it is an important defense mechanism. Experimental evidence supports this notion, insofar as intravenous injection of DNase in animals infected with bacteria or viruses increases bacteremia or viremia (5, 6), confirming that NETs act (at the very least) to prevent microorganism dissemination.

Despite the foremost role NETosis in neutrophil biology, host defense, and pathophysiology, the underlying molecular mechanisms remain only partially understood. Several studies have shown that endogenous reactive oxygen species (ROS) are needed for NET formation. Accordingly, some ROS (e.g., singlet oxygen, HOCl, H_2_O_2_) can directly induce NETs in neutrophils (7–10). More direct evidence is that inhibiting either NADPH oxidase or myeloperoxidase prevents NET formation in response to PMA or bacteria (7, 9–11). Similarly, neutrophils from chronic granulomatous disease patients, which are unable to generate ROS (12), fail to undergo NETosis in response to PMA (7). As a result, it has become widely accepted that NETosis is a ROS-dependent process. This is consistent with the fact that most of the studies on NETosis have employed PMA, a powerful NADPH oxidase activator. However, the phenomenon is also known to occur in response to stimuli that are ineffective ROS inducers, such as calcium ionophores, GM-CSF, TNFα, or IL-1β (11, 13), which begs for the issue to be revisited.

Arginine deimination has emerged as another potential underpinning of NETosis, insofar as citrullinated proteins, PAD2, and PAD4 associate with NETs in response to inflammatory stimuli in humans (14, 15). In addition, pretreatment of human neutrophils with the general PAD inhibitor, chloraminidine, was found to hinder NETosis (16–21). However, the actual PAD isoform responsible for this effect has yet to be identified in human neutrophils, even though studies conducted in knockout animals have suggested PAD4 as the main citrullinating enzyme (17–19). The recent availability of a selective PAD4 inhibitor, GSK484 (22), at last offers an opportunity to further explore the matter in human neutrophils.

The intracellular signaling pathways acting upstream of NETosis have also begun to be elucidated. However, the overall picture remains blurred, as it mostly consists of isolated observations concerning individual pathways, made using different stimuli, and using different methods. Thus, the Syk and PI3K pathways appear to be crucial in neutrophils stimulated by PMA, inflammatory crystals, or β-glucan (13, 23–27), but Syk seems to be dispensable for NETosis triggered by FcγRIIIb clustering (28). For p38 MAPK, Behnen et al. reported that it is needed for NET formation induced by immobilized immune complexes (26), but other investigators found no involvement using different stimulatory conditions (29, 30). Similarly, MEK was reported to control NETosis in response to FcR engagement or calcium pyrophosphate crystals (13, 23–28) but little is known about soluble stimuli. In the case of PKC, it was reported to be necessary for NETosis elicited by PMA or oxidized LDL (28, 31, 32), but not in response to mercury-containing compounds (30). Finally, one group reported that JNK is required for NETosis in cells stimulated by PMA, LPS, or bacteria (33) while another group showed that TAK1 can control NET formation in response to FcRIIIB clustering (13, 23–27). In summary, much remains to be done to sort, complete, and integrate the available information.

Finally, current methodological approaches to quantify NETs suffer from significant drawbacks, in particular the inclusion of an abundant non-specific signal. Here, we describe a NET quantification approach based on novel fluorescent polymers that only bind extruded chromatin. This allows for a specific, reliable, standardized quantification of NETosis, and was applied to decipher some of the underlying mechanisms, as well as the upstream signaling pathways controlling the phenomenon.

## Materials and methods

### Antibodies and reagents

Antibodies against myeloperoxidase (A0398) were from Dako/Agilent (Mississauga, ON, Canada); antibodies against citrullinated histone H3 were from Abcam (Ab5103); phospho antibodies were from Cell Signaling (Beverly, MA, USA). Ficoll-Paque Plus was from GE Biosciences (Baie d'Urfé, Qc, Canada); endotoxin-free (< 2 pg/ml) RPMI 1640 was from Wisent (St-Bruno, Qc, Canada). Recombinant human cytokines were from R&D Systems (Minneapolis, MN, USA). Actinomycin D, cycloheximide, N-formyl-methionyl-phenylalanine (fMLP), and phenylmethanesulphonyl fluoride (PMSF) were from Sigma (St. Louis, MO, USA). Kinase inhibitors and fluorescent probes were all purchased through Cedarlane Labs (Mississauga, Canada). PlaNET reagents, fluorescent chromatin-binding polymers, were from Sunshine Antibodies (https://sunshineantibodies.com/planet-001.html).

### Cell isolation and culture

Neutrophils were isolated from the peripheral blood of healthy donors, under a protocol approved by an institutional ethics committee (Comité d'éthique de la recherche du CIUSS de l'Estrie-CHUS). All subjects gave written informed consent in accordance with the Declaration of Helsinki. Briefly, whole blood was collected using an anticoagulant (sodium citrate), and successively submitted to dextran sedimentation, Ficoll separation, and water lysis—as previously described (34) The entire procedure was carried out at room temperature and under endotoxin-free conditions. As determined by Wright staining and FACS analysis, final neutrophil suspensions contained fewer than 0.1% monocytes or lymphocytes; neutrophil viability exceeded 98% after 4 h in culture, as determined by trypan blue exclusion and by Annexin V/propidium iodide FACS analysis.

### NETosis assays

For each condition, a 500-μl drop of a neutrophil suspension (2 × 10^6^/ml in RPMI 1640/2% autologous serum) was deposited onto coverslips that were freshly coated with poly-L-lysine, and the cells were left to adhere for 60 min in a cell culture incubator. Inhibitors and/or stimuli were then added and the final volume brought to 550 μl, prior to a 4-h incubation (37°C, 5% CO_2_). Reactions were stopped by adding 500 μl ice-cold PBS containing 1 mM PMSF, and the coverslips were placed on ice for 10 min. At this point, one of two procedures were followed.

When antibodies were used for NET detection, the liquid on the coverslips was discarded and cells were fixed for 15 min in ice-cold PBS containing 2% parafornaldehyde, as well as a nuclear stain (e.g., DAPI, Hoechst 33342). The fixed cells were then washed with ice-cold PBS, and blocked for 60 min with PBS containing 5% normal goat serum (i.e., serum from the same species in which the 2nd antibody was raised), hereafter referred to as Blocking Buffer. Cells were next incubated in PBS containing the primary antibody (anti MPO, 1:1,000) for 90 min, washed, and incubated 45 min with an Alexa 568-labeled secondary Ab (goat anti-rabbit, Molecular Probes #A11011, 1:2,000) in Blocking Buffer. Coverslips were then mounted onto glass slides using a drop of mounting medium (ProLong Gold, Life Technologies) and sealed, prior to epifluorescence microscopy analysis.

When PlaNET reagents were used for NET detection, the liquid on the coverslips was discarded and cells were incubated (90 min on ice, with gentle shaking) in 1 ml of PBS containing 1 mM PMSF and diluted PlaNET reagent (as recommended by the manufacturer). Cells were finally fixed (15 min, room temperature) in PBS containing 2% parafornaldehyde, as well as a nuclear stain. The fixed cells were washed once with PBS, and the coverslips were mounted as described above, and analyzed by epifluorescence microscopy. For quantitation, 3 fields at 10x magnification were counted, that never included the coverslip edges; this amounts to about 1,000 neutrophils in total.

### Immunoblots

Samples were prepared, electrophoresed, transferred onto nitrocellulose, and processed for immunoblot analysis as previously described (35, 36).

### Data analysis

All data are represented as mean ± SEM. Unless otherwise stated, statistical differences were analyzed by Student's *t*-test for paired data, using Prism 7 software (GraphPad, San Diego, CA, USA).

## Results

### A new approach to visualize and quantify NET formation

**Figure 1:**
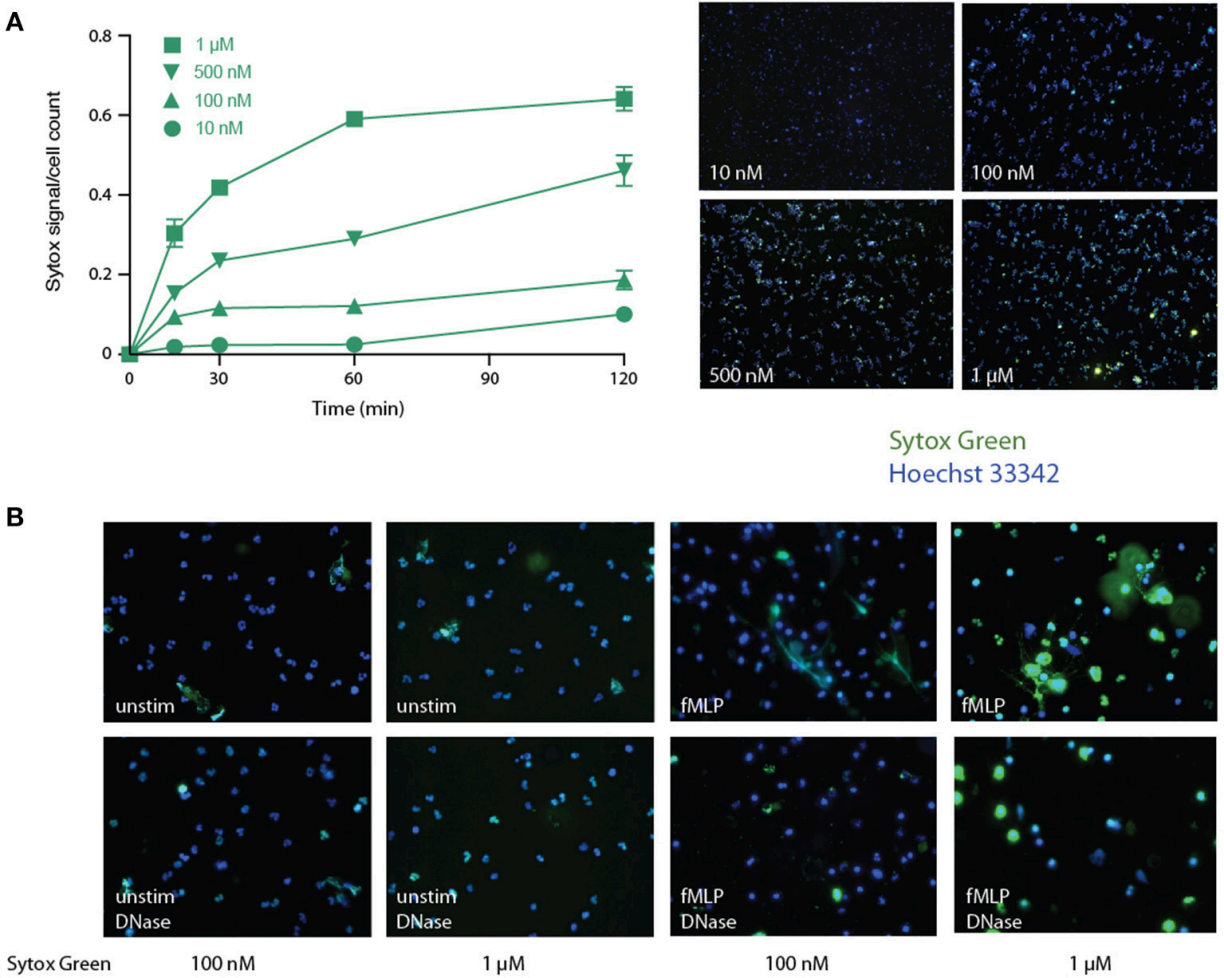
Detection of NETs using Sytox Green in human neutrophils. **(A)** Unstimulated neutrophils were cultured in suspension for the indicated times in the presence of increasing concentrations of Sytox Green, prior to the addition of Hoechst 33342 and subsequent fluorescence microscopy analysis of unfixed cells. Total Sytox Green fluorescence values were divided by total Hoechst 33342 fluorescence, to normalize for cell number. Mean ± s.e.m. from duplicate measurements for each experimental condition from a representative experiment, shown on the right at 10X magnification. **(B)** Neutrophils were cultured for 4 h on poly-L-lysine coated coverslips with the indicated Sytox Green concentrations, in the absence (“ctrl”) or presence of 30 nM fMLP. The cells were then digested or not with DNase I (100 U/ml, 30 min, 37°C), then stained with Hoechst 33342, prior to fluorescence microscopy analysis. A representative experiment is shown (at 40X magnification).

Various procedures have been used for this purpose, yet they all suffer significant drawbacks, not the least of which is the inclusion of a false positive fluorescence signal. A widespread approach is to incubate neutrophils with a DNA dye (e.g., Sytox Green) that is described as cell-impermeable by its manufacturer, and to analyze total fluorescence in the supernatants. However, we observed that over a concentration range that is far inferior to commonly used (i.e., 5–10 μM) Sytox Green concentrations (31, 33, 37–39), the dye rapidly and dose-dependently leaks into living cells (Figure 1A). A notable effect was consistently detected after only 15 min using just 100 nM of the dye, and massive leaking was observed using 1 μM by 30 min in unstimulated cells (Figure 1A). This was not due to non-specific staining by Sytox Green of DNA from necrotic cells, since the latter were undetectable at short incubation times, as determined by a lack of PI staining (not shown). Neither did the cell-associated Sytox Green fluorescence result from its staining extracellular DNA, as few NETs were ever observed in unstimulated cells, and accordingly, virtually all the fluorescent signal was still present following DNase I digestion under these conditions (Figure 1B, left panels). In fMLP-stimulated cells, some NETs were observed using Sytox Green, as expected, but much of the extracellular signal was not associated with extruded DNA, as it was impervious to DNase treatment (Figure 1B, right panels). Thus, the use of Sytox Green entails a large, cell-permeable, non-specific signal that cannot be easily distinguished from NET-associated fluorescence (unless DNase-treated samples are always processed in parallel).

**Figure 2:**
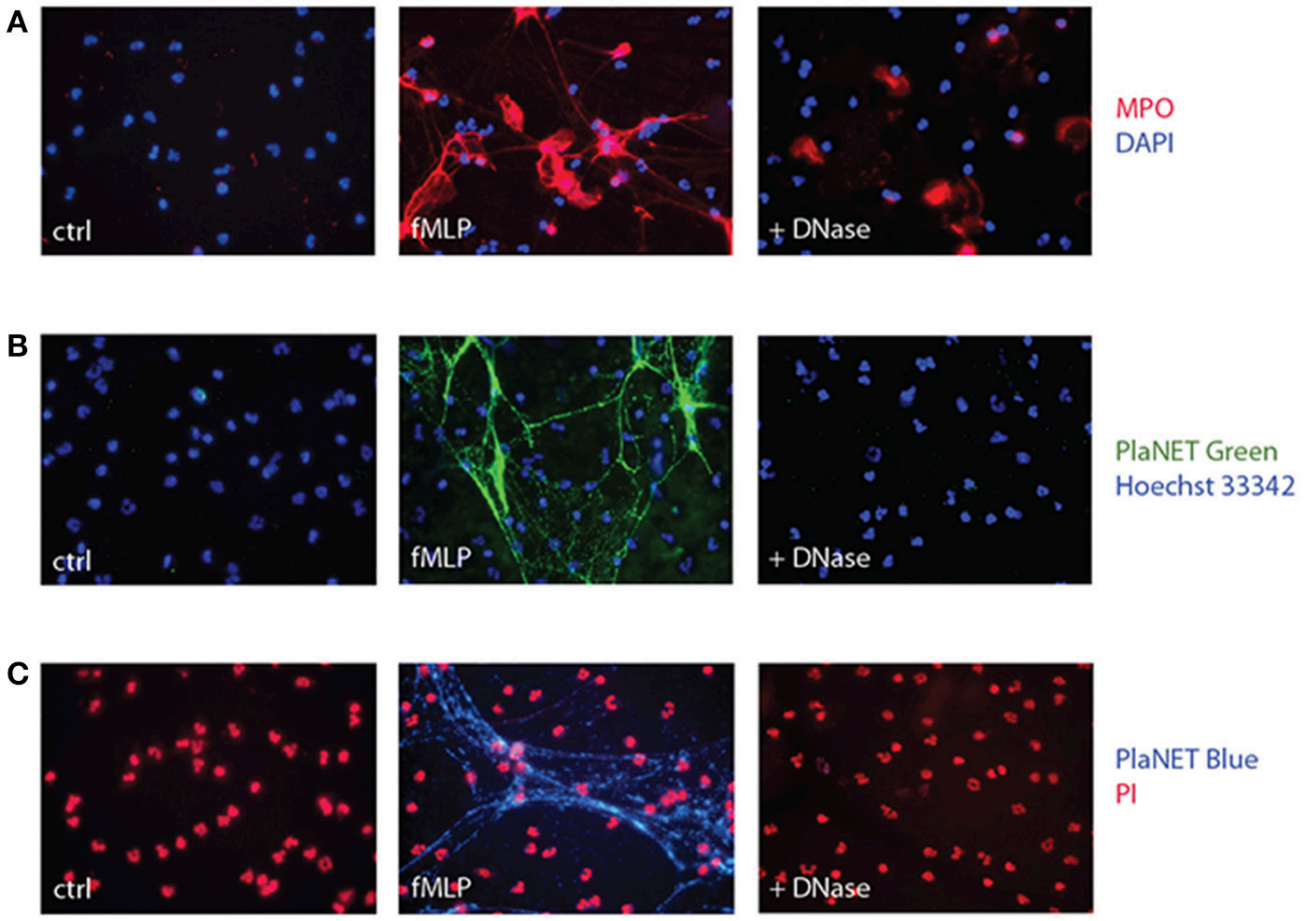
Detection of NETs using MPO or PlaNET reagents in human neutrophils. **(A)** Neutrophils cultured on poly-L-lysine-coated coverslips were incubated for 4 h in the absence (“ctrl”) or presence of 30 nM fMLP, and digested or not with DNase I, prior to fluorescent microscopy detection of MPO as described in *Methods*. A representative experiment is shown (40X magnification). **(B)** Neutrophils cultured on poly-L-lysine-coated coverslips were incubated for 4 h in the absence (“ctrl”) or presence of 30 nM fMLP, and further incubated in the presence or absence of DNase I (100 U/ml, 30 min, 37°C), prior to fluorescent microscopy detection of NETs using PlaNET Green and Hoechst 33342 counterstaining, as described in *Methods*. A representative experiment is shown (40X magnification). **(C)** Neutrophils were treated as described in **(B)**; fluorescent microscopy detection of NETs was conducted using PlaNET Blue and propidium iodide counterstaining (5 μM, 20 min), as described in *Methods*. A representative experiment is shown (40X magnification).

Another common approach is to stain NETs using antibodies directed against associated proteins. However, this can be misleading as several such proteins (e.g., MPO, elastase) readily associate with cell membranes upon their release from the cells (40, 41). And indeed, an important fluorescence signal remains near the cell surface following DNase digestion of NETs when the latter are detected using anti-MPO Abs (Figure 2A). This was not due to residual background staining, since no second antibody fluorescence (Alexa 568) was detectable when the experiment was repeated using an isotype control rabbit antibody in substitution for the anti MPO primary antibody (Figure S1). Thus, commonly used approaches based on the detection of NET-associated granule proteins, or on Sytox Green staining, are fraught with complications when total fluorescence is counted (as is usually the case), as it includes a substantial (and often predominant) non-specific signal.

In an attempt to overcome this shortcoming, we resorted to PlaNET reagents—newly developed NET detection reagents that are based on fluorescent, chromatin-binding polymers. As shown in Figures 2B,C, PlaNET reagents strongly stain NETs in activated cells, and DNase I digestion completely obliterates the PlaNET reagent signal, thereby showing that it is strictly extracellular. In agreement with these findings, PlaNET fluorescnce was also undetectable in cells that were deliberately made necrotic (Figure S2). Thus, measuring total PlaNET reagent fluorescence proves to be a straightforward and specific way of assessing NETosis, independently of necrosis. To ensure optimal comparisons between samples and experiments, PlaNET fluorescence can be standardized. To this end, we developed a Java-based plug-in (available at http://mcdonaldlab.co.nf/McDonald_Lab/plugin.html) that counts total PlaNET fluorescence and divides it by the number of events (i.e., cells) in the fluorescence channel used for the DNA counterstain, yielding standardized NETosis values.

### Induction of NET generation by various stimuli, and signaling pathways involved

**Figure 3:**
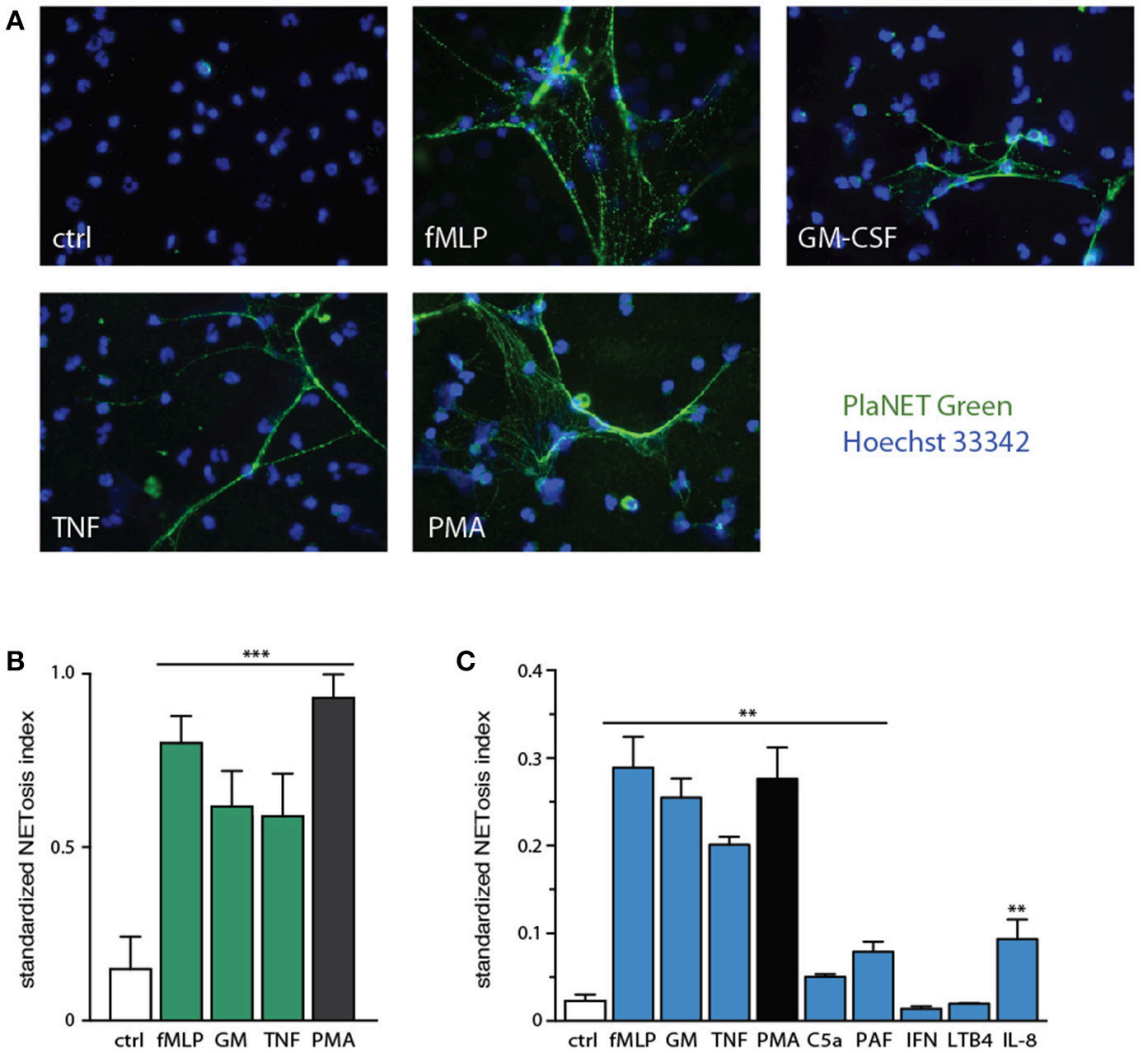
Relative potency of physiological neutrophil agonists or PMA to induce NETosis. **(A)** Neutrophils cultured on poly-L-lysine-coated coverslips were incubated for 4 h in the absence (“ctrl”) or presence of 30 nM fMLP, 1 nM GM-CSF, 100 U/ml TNFα, or 50 nM PMA. NETosis was then assessed using PlaNET Green as described in *Methods*. Representative fields are shown at 40X magnification. **(B)** Quantitative representation of the above experiments, in which PlaNET Green fluorescence values were standardized according to total cell number (i.e., the number of individual events detected using a cell-permeable nuclear stain), thus yielding a NETosis index. Mean ± s.e.m. from at least 5 independent experiments. ^***^p < 0.001 vs. unstimulated cells. **(C)** Neutrophils were cultured as described above for 4 h in the absence (“ctrl”) or presence of 30 nM fMLP, 1 nM GM-CSF, 100 U/ml TNFα, 50 nM PMA, 30 nM C5a, 50 nM PAF, 100 U/ml IFNγ, 100 nM LTB4, or 10 nM IL-8. Quantitative representation of these experiments, in which PlaNET Blue fluorescence values were standardized as described in **(B)**. Mean ± s.e.m. from 3 independent experiments. ^**^p < 0.02 vs. unstimulated cells.

We used this standardized approach to assess NETosis induction by various neutrophil agonists. As shown in Figure 3A, few unstimulated neutrophils generate NETs after a 4-h incubation period, whereas exposure to various physiological stimuli, or to PMA, results in abundant NET formation. By standardizing NETosis using the Java plug-in, we could compare the relative ability of the stimuli to induce this response; fMLP and PMA stood out as the most potent inducers, with TNFα and GM-CSF following not far behind (Figure 3B), though differences among these stimuli were not found to be statistically significant by one-way ANOVA analysis. Several other physiological stimuli (namely, C5a, PAF, IL-8) were also found to promote NET formation, albeit less potently (*p* < 0.01 using one-way ANOVA with Dunnett's correction) than fMLP, GM-CSF, or TNF (Figure 3C); in these experiments, we used PlaNET Blue, as it offers an even better signal-to-noise ratio than PlaNET Green. Finally, other neutrophil stimuli (e.g., LTB4, IFNγ) failed to stimulate NETosis altogether (Figure 3C).

**Figure 4:**
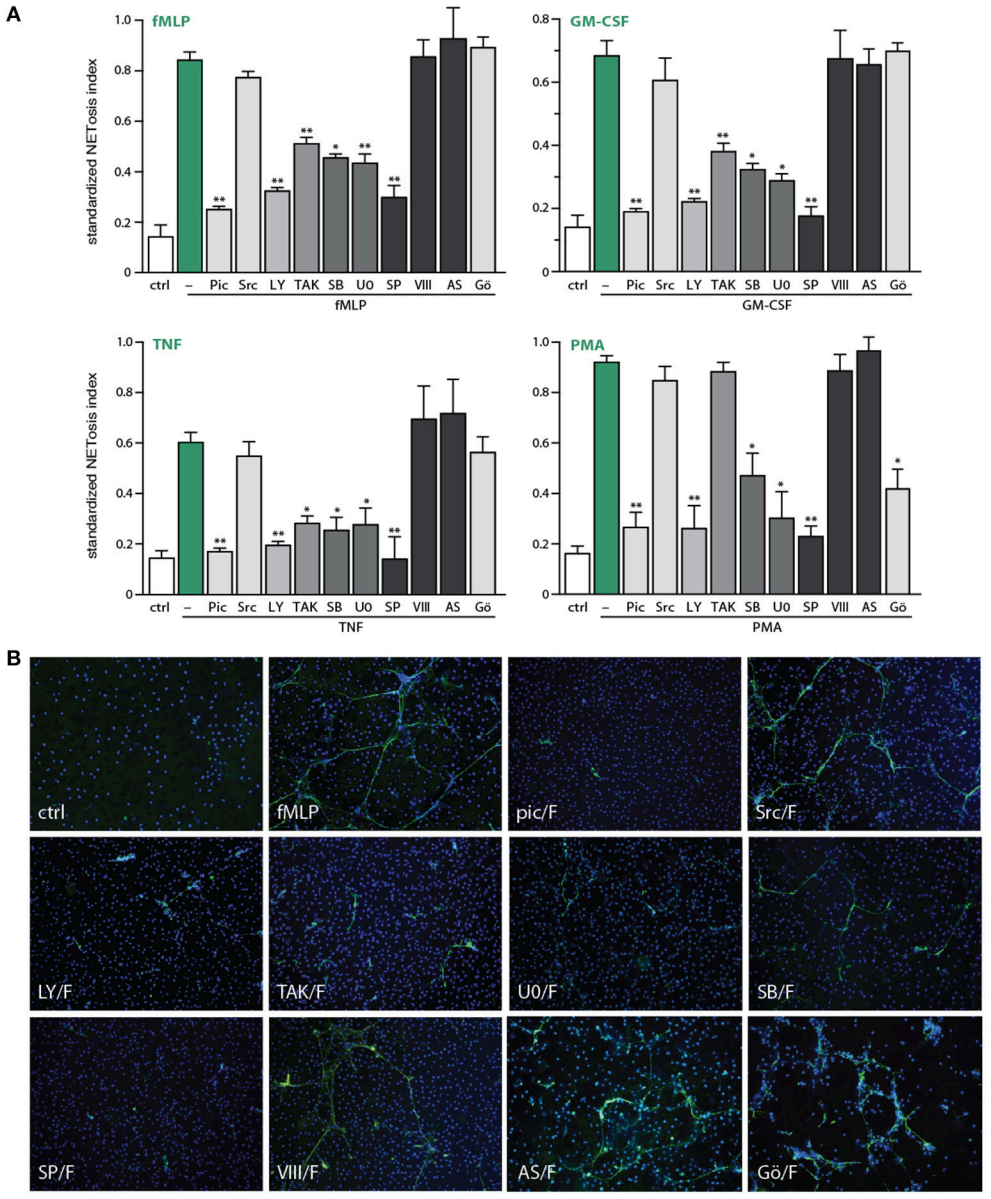
Signaling pathways controlling NETosis induced by physiological neutrophil agonists or PMA. Neutrophils cultured on poly-L-lysine-coated coverslips were pre-treated (15 min, 37°C) with the following inhibitors or their diluent (DMSO): 10 μM piceatannol (Syk inhibitor); 10 μM SrcI1 (Src family kinase inhibitor); 10 μM LY294002 (PI3K inhibitor); 1 μM (5Z)-7-oxozeaenol (TAK1 inhibitor); 1 μM SB202190 (p38 MAPK inhibitor); 10 μM U0126 (MEK inhibitor); 10 μM SP600125 (JNK inhibitor); 10 μM JNK inhibitor VIII (a different JNK inhibitor); 5 μM AS601285 (a third JNK inhibitor); 10 μM Gö6976 (a PKC inhibitor). The cells were then further incubated for 4 h in the absence (“ctrl”) or presence of 30 nM fMLP, 1 nM GM-CSF, 100 U/ml TNFα, or 50 nM PMA. NETosis was assessed using PlaNET Green as described in *Methods*. **(A)** Quantitative representation of these experiments, expressed as NETosis index. Mean ± s.e.m. from at least 3 independent experiments. ^*^p < 0.05 and ^**^p < 0.01 vs. stimulus alone. **(B)** Representative fields for each experimental condition are shown at 10X magnification.

We next blocked discrete signaling intermediates using selective inhibitors, prior to stimulation with physiological agonists or PMA, to identify which pathways control NET generation. As shown in Figure 4A, inhibitors of TAK1, MEK, or p38 MAPK potently hindered NETosis in response to GM-CSF, fMLP, or TNFα. In the case of PMA-elicited NETosis, MEK and p38 MAPK inhibition also affected this response, but TAK1 inhibition failed to do so—in keeping with the fact that PMA does not activate TAK1 in neutrophils (our unpublished data). Accordingly, the PMA-induced phosphorylation of ERK, p38 MAPK and Akt were similarly unaffected by TAK1 inhibition (Figure S3). By comparison, inhibition of the Syk, PI3K, and JNK pathways nearly or completely abrogated NET formation in response to all agonists tested (Figure 4A). Finally, inhibition of Src tyrosine kinases consistently failed to interfere with NETosis (Figure 4A). Likewise, inhibition of PKC impaired NETosis elicited by PMA, as expected, but not in response to physiological stimuli (Figure 4A).

In addition to the above quantitative data, direct microscopic observation revealed qualitative differences between the effects of the various inhibitors toward NETosis. As shown in Figure 4B, inhibition of TAK1, MEK, or p38 MAPK yielded much shorter extracellular chromatin filaments, with little or no interconnectivity, whereas chromatin extrusion *per se* was clearly less affected. This suggests that chromatin extrusion and filament elongation and/or branching could represent distinct steps in the NETosis process. By comparison, cell pretreatment with inhibitors of Syk or PI3K, or the JNK inhibitor, SP600125 (Figure 4B), resulted in little or no chromatin extrusion, suggesting that they prevent this step (or perhaps upstream events). The case of JNK inhibition was particularly intriguing, given that some of the stimuli used (e.g., fMLP, GM-CSF, PMA) do not promote the phosphorylation of JNK in neutrophils, and can therefore hardly induce neutrophil responses by acting via this kinase. To ensure that the inhibition of NETosis by SP600125 cannot be attributed to non-specific effects, we used a potent and structurally unrelated JNK inhibitor (called JNK inhibitor VIII). As shown in Figure 4A (last bar) and Figure 4B (last pane), NETosis was unaffected using this second JNK inhibitor, for all stimuli tested. Similarly, a third JNK inhibitor, AS601245, also failed to affect NETosis (Figure 4A). Together, these observations make it very unlikely that JNK participates in controlling NETosis.

**Figure 5:**
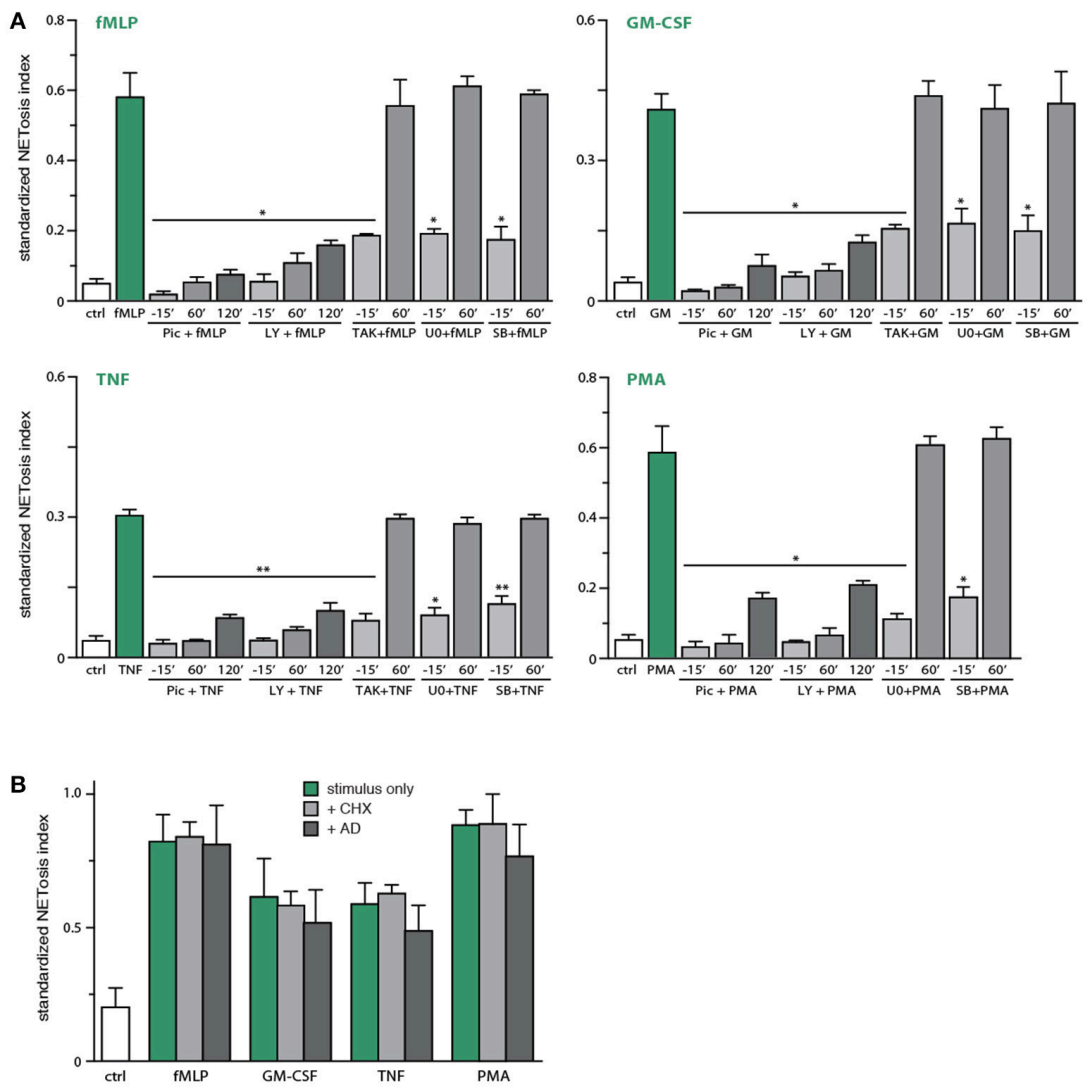
Identification of early and late processes underlying NET generation. **(A)** Neutrophils cultured on poly-L-lysine-coated coverslips were treated either before or after stimulation for the indicated times with the following inhibitors or their diluent (DMSO): 10 μM piceatannol (“pic,” Syk inhibitor); 10 μM LY294002 (PI3K inhibitor); 1 μM (5Z)-7-oxozeaenol (TAK1 inhibitor); 1 μM SB202190 (p38 MAPK inhibitor); 10 μM U0126 (MEK inhibitor). The cells were also stimulated for 4 h in the absence (“ctrl”) or presence of 30 nM fMLP, 1 nM GM-CSF, 100 U/ml TNFα, or 50 nM PMA. NETosis was then assessed using PlaNET Green as described in *Methods*. Quantitative representation of these experiments, expressed as NETosis index. Mean ± s.e.m. from 3 independent experiments. ^*^p < 0.05 and ^**^p < 0.01 vs. stimulus alone. **(B)** Neutrophils cultured on poly-L-lysine-coated coverslips were pre-treated (15 min, 37°C) with 20 μg/ml cycloheximide, 5 μg/ml actinomycin D, or their diluent (DMSO), prior to a further incubation of 4 h in the absence (“ctrl”) or presence of 30 nM fMLP, 1 nM GM-CSF, 100 U/ml TNFα, or 50 nM PMA. NETosis was then assessed using PlaNET Green as described in *Methods*. Quantitative representation of these experiments, expressed as NETosis index. Mean ± s.e.m. from at least 3 independent experiments.

Because Syk and PI3K emerged as important upstream intermediates controlling NETosis, we next investigated whether this reflects early or late signaling events, given that NETosis requires 3–4 h to be effectively detected. To this end, kinase inhibitors were either added 15 min before neutrophil stimulation, or 60–120 min afterwards. As shown in Figure 5A, NET formation was effectively prevented even when the Syk and PI3K inhibitors were added 120 min post-stimulation, indicating that these pathways are mobilized late in the NETosis phenomenon. Similar results were obtained using SP600125 (Figure S4), though this likely reflects off-target effects, as explained above. By contrast, addition of TAK1, p38 MAPK, or MEK inhibitors 60–120 min after neutrophil stimulation failed to affect NET formation (Figure 5A and data not shown), indicating that the contribution of these kinases occurs early in the induction of NETosis. Because stimulated neutrophils express several cytokine and chemokine genes (and release the corresponding proteins) in the same time frame required for NET formation, and since several such products are NET inducers, we also examined whether gene transcription or *de novo* protein synthesis might participate in NETosis. As shown in Figure 5B, the blockade of transcription (using actinomycin D) or of protein synthesis (using cycloheximide) failed to alter NETosis elicited by physiological stimuli or PMA.

### Involvement of endogenous ROS and PAD4 in NET generation

**Figure 6:**
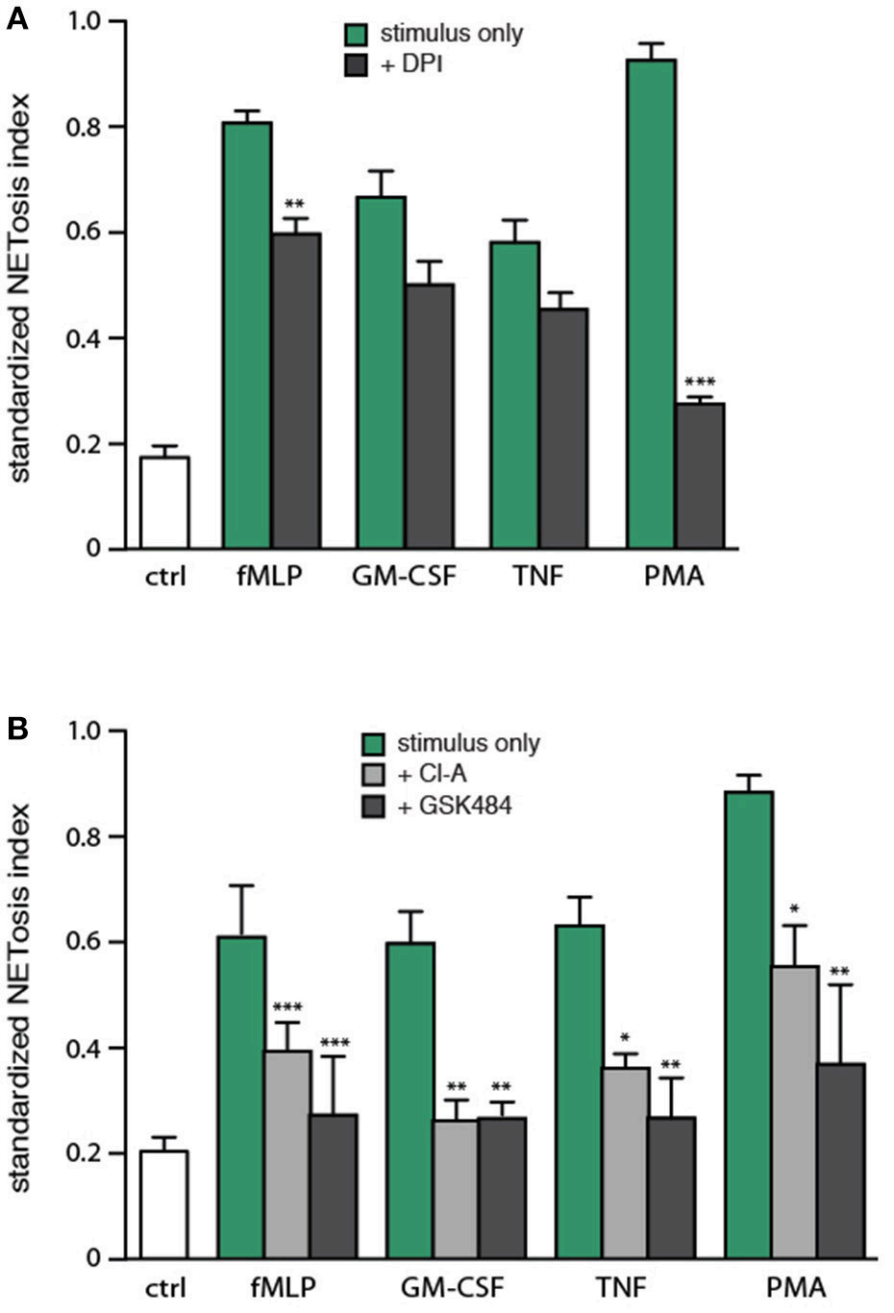
Involvement of endogenous ROS and PAD4 in NET generation. **(A)** Neutrophils cultured on poly-L-lysine-coated coverslips were pre-treated (15 min, 37°C) with 10 μM DPI or its diluent (DMSO), prior to a further incubation of 4 h in the absence (“ctrl”) or presence of 30 nM fMLP, 1 nM GM-CSF, 100 U/ml TNFα, or 50 nM PMA. NETosis was then assessed using PlaNET Green as described in *Methods*. Quantitative representation of these experiments, expressed as NETosis index. Mean ± s.e.m. from at least 5 independent experiments. ^**^p < 0.01 and ^***^p < 0.0001 vs. stimulus alone. **(B)** Neutrophils cultured on poly-L-lysine-coated coverslips were pre-treated (15 min, 37°C) with 10 μM chloraminidine (“Cl-A,” a general PAD inhibitor), 10 μM GSK484 (a PAD4 inhibitor), or their diluent (DMSO), prior to a further incubation of 4 h in the absence (“ctrl”) or presence of the above stimuli, followed by determination of the NETosis index. Mean ± s.e.m. from at least 4 independent experiments. ^*^p < 0.04, ^**^p < 0.01, and ^***^p < 0.001 vs. stimulus alone.

Because NETosis can be induced by stimuli that are ineffective ROS inducers (11, 13), it would seem that under some circumstances, NETosis must take place independently of ROS production. To investigate the issue, neutrophils were pretreated with DPI (a NADPH oxidase inhibitor), prior to stimulation. As expected, PMA-elicited NETosis was almost entirely dependent on NADPH oxidase activation (Figure 6A). NET formation in response to fMLP was also significantly affected by DPI, but to a far lesser extent (Figure 6A). In contrast, TNF- or GM-CSF-induced NETosis were not significantly inhibited by DPI (Figure 6A). These results show that the phenomenon is largely ROS-independent in response to various physiological agonists.

Arginine deimination has also been proposed to participate in NETosis, insofar as PAD inhibition or deficiency hinders NETosis (16–19). To further investigate the role of PAD4 in NETosis, we pretreated neutrophils with either chloraminidine (a general PAD inhibitor) or GSK484 (a selective PAD4 inhibitor) (22), prior to stimulation with physiological agonists or PMA. Both inhibitors abrogated the citrullination of histone H3, as expected (Figure S5). As shown in Figure 6B, both chloraminidine and GSK484 strongly inhibited NET formation under all conditions tested. NETosis therefore appears to involve PAD4 in human neutrophils. Worthy of note is that chloraminidine did not retain its potency for more than a month after having been prepared; care should therefore be taken to use only freshly-prepared stocks of this inhibitor.

## Discussion

Since its discovery a dozen years ago, NETosis has emerged as a major neutrophil functional response, and its study represents an area of intense ongoing research. Whereas diverse methods have been used to assess NETosis, most suffer from significant drawbacks, in particular the inclusion of a false positive signal that can often be predominant. In this study, we used newly developed fluorescent reagents that allow a streamlined, reliable, and standardized assessment of NET formation. This allowed us to quantitatively compare NETosis induction by various physiological stimuli, to shed a new light on the signaling pathways involved, and to unveil some of the underlying mechanisms.

Using PlaNET reagents, we showed that NETosis can be measured specifically, insofar as the entire signal disappears following DNase I digestion—unlike widely used assays based on the detection of NET-associated proteins (such as myeloperoxidase or elastase), in which a strong fluorescent signal remains after DNase treatment, possibly reflecting the propensity of several neutrophil granule proteins to strongly associate with cell membranes upon their release from the cells (40, 41). Likewise, PlaNET reagents do not enter live or necrotic cells, unlike widely used detection reagents such as Sytox Green, which do so rapidly and in a concentration-dependent manner. A workaround for the shortcomings of conventional approaches to assess NETosis would be to always carry out experiments in the presence and absence of DNase digestion, even though this would automatically double the size and cost of any experiment. Thus, the specificity of PlaNET reagents represents a major advantage over commonly used approaches to study NETosis. This being said, PlaNET reagents are not without some drawbacks, as we found that they are not completely suitable for kinetic assessment of NETosis in microtiter plate assays. This is because neutrophils co-incubated with PlaNET reagents somehow ingest some of the polymers, resulting in a non-specific signal. This could be prevented by including PMSF in the culture medium, but whether incubation of living cells in the continued presence of this inhibitor might affect other processes would be a potential concern. For this reason, we would not advise using PlaNET reagents for kinetics studies in a plate reader.

We also developed a simple Java plug-in to standardize NETosis measurement based on the total number of neutrophils, and found that doing so helps minimize both intra-experiment and inter-donor variation. In this regard, another group recently reported similar benefits from standardizing NET detection (42), though they used Sytox Green which we (and other investigators) found to enter the cells to a significant degree. Another important benefit of standardizing is that it allowed us to quantitate the relative ability of various stimuli to elicit NETosis, and the extent to which various inhibitors affect the phenomenon. Thus, we found that fMLP, PMA, TNF, and GM-CSF are potent inducers; IL-8 are also a good inducer, but comparatively weaker; finally, C5a and PAF proved to be weak stimuli. Conversely, certain neutrophil activators were found to induce little or no NET formation (e.g., LTB4, IFNγ), showing that not all neutrophil stimuli act as NET inducers.

Pharmacological blockade of various signaling pathways revealed that several kinases (e.g., Syk, PI3K, TAK1, p38 MAPK, MEK) profoundly affect NETosis. Although Syk and PI3K inhibitors were consistently more effective than the ones for TAK1 and the MAP kinases, this difference was not found to be statistically significant by one-way ANOVA analysis. By contrast, inhibition of Src tyrosine kinases consistently failed to interfere with NETosis. For physiological stimuli such as TNFα, fMLP, or GM-CSF, our data are consistent with our previous findings, which showed that they can all signal through the TAK1-MEK or TAK1-p38 axes in neutrophils (43, 44). Conversely, PMA does not activate TAK1 in these cells (our unpublished data), and accordingly, TAK1 inhibition had no significant effect on PMA-elicited NETosis. In the particular case of JNK inhibition, we found it surprising that in TNF-stimulated neutrophils, TAK1 inhibition of NETosis was less pronounced than that exerted by the widely used JNK inhibitor, SP600125, given that TNF activates JNK downstream of TAK1 in these cells (43). Likewise, we found it peculiar that SP600125 should abrogate NETosis even when ineffective JNK activators (e.g., PMA, fMLP, GM-CSF) were used as stimuli. In this regard, SP600125 is known to exert non-specific effects toward 13 other kinases (45), and to even inhibit PI3K as effectively as wortmannin in mast cells (46). The latter observation is particularly alarming, in view of how potently PI3K inhibition prevents NETosis, as shown herein and in other studies (47, 48). Together, the above considerations cast a serious doubt as to whether SP600125 affects NETosis through JNK inhibition, as opposed to off-target actions. To settle the matter, we resorted to very selective, structurally unrelated JNK inhibitors (i.e., JNK inhibitor VIII and AS601285), which both failed to affect NETosis in response to all stimuli tested. This is compelling evidence that JNK does not control the phenomenon. This conclusion contrasts with a recent study, in which SP600125 and TCSJNK6o (also known as JNK inhibitor VIII) obliterated LPS-induced NETosis while they only minimally affected the phenomenon in PMA-treated cells, using a Sytox Green-based NET assay (33). This discrepancy between their data and ours is not easy to resolve, especially since we used similar concentrations of PMA and JNK inhibitors. However, the experimental procedures differ significantly. Because we thoroughly controlled for false positives (i.e., DNAse-insensitive or necrotic cell-derived signals) when assessing NETosis, and because we used three different JNK inhibitors, we stand by our conclusion, that JNK does not control NETosis in response to several classes of neutrophil stimuli.

Previous reports had already shown that Syk and PI3K are crucial for PMA-induced NETosis (47–49). Our data confirm these observations, but more importantly, reinforce their significance by demonstrating that this is also true of NETosis triggered by physiological stimuli. We further showed that Syk and PI3K do so by acting upon chromatin extrusion or upstream processes, and that this involves late signaling events in NETosis (occurring at about 120 min of stimulation). This is a major new observation. The nature of the late processes affecting NETosis, however, remains elusive. We could exclude newly-made cytokines and chemokines as potential candidates, even though they are produced in the right time frame and are potent NET inducers, since neither cycloheximide nor actinomycin D were found to affect NETosis in response to any of the stimuli used. Khan and colleagues similarly observed that cycloheximide does not affect NET formation in response to PMA or ionomycin, but reported that actinomycin D blocks the phenomenon (50). However, another group (51) found no effect of either cycloheximide or actinomycin D on NETosis elicited by PMA or *C. albicans*, in full agreement with our data. Thus, while it is quite clear that *de novo* protein synthesis does not contribute to NET formation, there is growing evidence for a similar conclusion in the case of gene transcription. In contrast to Syk and PI3K, other kinases (i.e., TAK1, p38 MAPK, MEK) seem to control early events (within the first 15 min) of the NETosis process, and to influence the length and degree of branching of extruded chromatin filaments, as opposed to chromatin extrusion itself. Studies are in progress to further define each aspect of the NETosis phenomenon.

We finally revisited the issue of whether NETosis is a ROS-dependent process. In this regard, it is noteworthy that the bulk of available data has been obtained using powerful NADPH oxidase activators, such as PMA or bacterial phagocytosis. However, NETosis has also been observed in response to stimuli that are ineffective ROS inducers, such as ionomycin, GM-CSF, TNFα, or IL-1β (11, 13). We confirmed herein that PMA-induced NETosis is indeed ROS-dependent (and PKC-dependent), but also show that NETosis occurring in response to various classes of physiologic stimuli is largely unaffected by inhibition of the NADPH oxidase or of PKC. These observations agree well with recent studies, which have shown that NETosis can take place in a ROS-independent fashion following neutrophil exposure to uric acid, mercury, nicotine, immune complexes, or endotoxin (20, 30, 42, 52, 53). Our data therefore adds to the mounting evidence that endogenous ROS are far from essential for NETosis, though they can certainly contribute to the process under some circumstances. By contrast, we found that NETosis occurring in response to all stimuli investigated (including PMA) depends on PAD4. Previous reports had reached a similar conclusion, based on the fact that chloraminidine prevents NET formation in response to calcium ionophores, bacteria, IL-8, PMA, or even nicotine (16, 20, 21). However, chloraminidine is a general PAD inhibitor that does not discriminate between PAD isoforms, and both PAD2 and PAD4 have been observed on NETs in a pathological setting in humans (15). Mouse studies have suggested that PAD4 might be the relevant molecule, insofar as NETosis does not occur in PAD4-deficient animals (17–19), whereas PAD2 is dispensable (54). Our finding, that a selective PAD4 inhibitor prevents NETosis as well as chloraminidine in human neutrophils, represents the first demonstration that PAD4 is also the relevant PAD isoform in humans. Thus, it appears that NETosis is a PAD4-dependent phenomenon that may also require endogenous ROS, depending on the stimulatory conditions. This represents a significant shift in how NETosis has heretofore been viewed.

In summary, we describe a reliable and specific appoach to assess NETosis. This allowed us to determine the relative potency of various physiologic NET inducers; to extend our knowledge of the signaling pathways involved, and of how they affect early or late stages of the phenomenon; and to identify PAD4 as required for NETosis, whereas ROS do not necessarily contribute to this response. In view of the involvement of NETs in several pathologies, our findings reveal potential molecular targets that could be exploited for therapeutic intervention. In this regard, inhibitors of several such molecules are already in phase I/II clinical trials (55–60).

## Author contributions

OT carried out all experiments, compiled all the data, and wrote the first draft. PM designed the research, mentored the other author, and wrote the final version of the paper.

### Conflict of interest statement

The authors declare that the research was conducted in the absence of any commercial or financial relationships that could be construed as a potential conflict of interest.
