# Supplementary material for: Physiological Stimuli Induce PAD4-Dependent, ROS-Independent NETosis, With Early and Late Events Controlled by Discrete Signaling Pathways
Source: Front Immunol. 2018 Sep 18;9:2036. doi: 10.3389/fimmu.2018.02036 (PMC6153332; doi:10.3389/fimmu.2018.02036)
Supplement: Supplementary file 1 [file Data_Sheet_1.PDF]

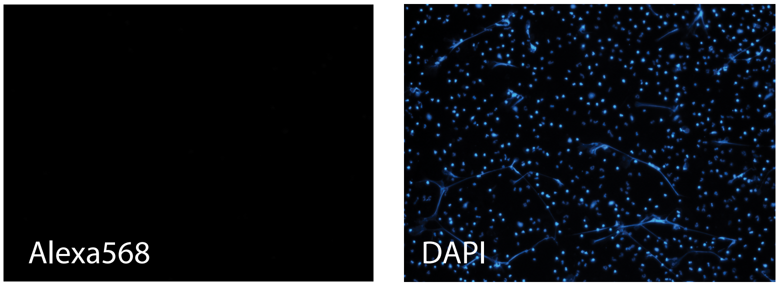

Figure S1. Isotope control experiment for MPO NET detection.
Neutrophils cultured on poly-L-lysine-coated coverslips were incubated for 4 h in the presence of 30 nM fMLP. Fixed cells were blocked with PBS containing 5% normal goat serum and DAPI, incubated with rabbit IgG at the same concentration as the anti-MPO rabbit IgG, and further incubated with goat anti-rabbit-Alexa 568, prior to fluorescent microscopy analysis. A representative experiment is shown (10X magnification).

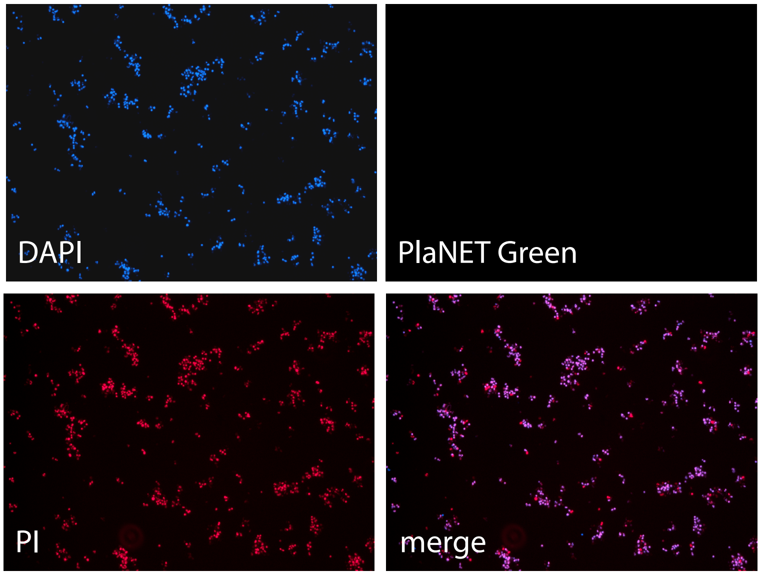

Figure S2. PlaNET reagents do not stain necrotic cells.
Neutrophils were cultured overnight without stimulation in 6-well plates, and cells that detached from the plates (i.e. necrotic cells) were collected. Necrotic cells were processed following the PlaNET Green procedure described in Methods, with the exception that propidium iodide was included (in addition to DAPI) during the final fixation step, to confirm that the cells were indeed necrotic. A representative experiment is shown (10X magnification).

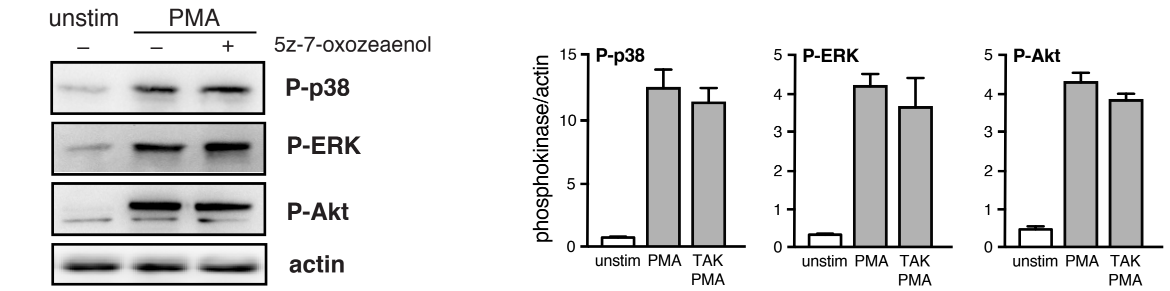

Figure S3. PMA-induced MAP kinase phosphorylation is TAK1-independent. Neutrophils were stimulated with 50 nM PMA for 10 min, prior to immunoblot analysis of their phospho-p38 MAPK, phospho-ERK, and phospho-Akt content. The same samples were also migrated on a parallel gel and immunoblotted for β-actin as a loading control. A representative experiment is shown, as well as compiled densitometric data (mean ± s.e.m.) from 3 independent experiments.

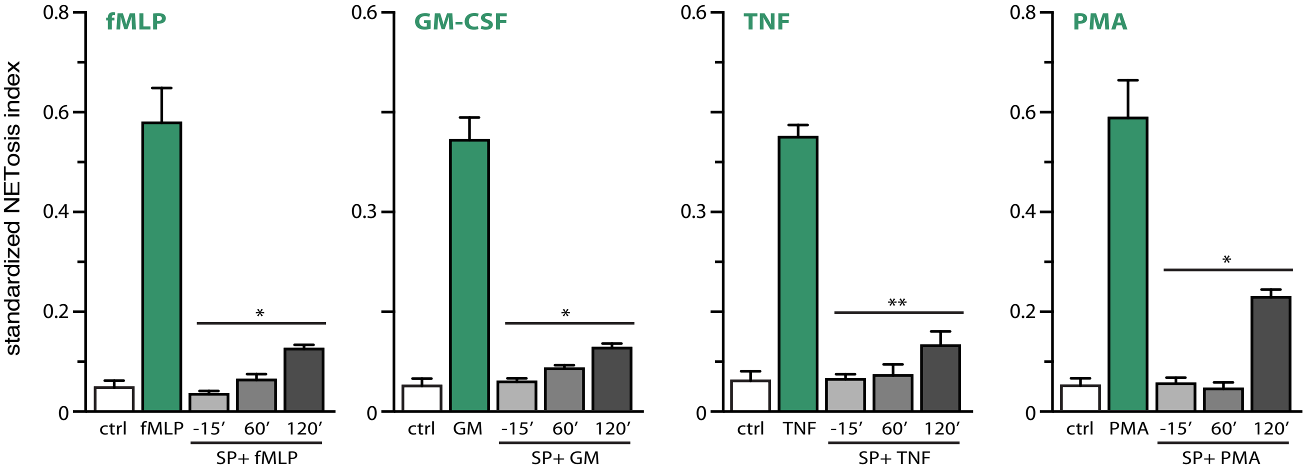

Figure S4. Involvement of late endogenous processes in NET generation. Neutrophils cultured on poly-L-lysine-coated coverslips were pre-treated for the indicated times with 10 μM SP600125 (JNK inhibitor) or its diluent (DMSO). The cells were then further incubated for 4h in the absence ("ctrl") or presence of 30 nM fMLP, 1 nM GM-CSF, 100 U/ml TNFα, or 50 nM PMA. NETosis was then assessed using PlaNET Green as described in Methods. Mean ± s.e.m. from 3 independent experiments. *, $p<0.03$; **, $p<0.006$ vs stimulus alone.

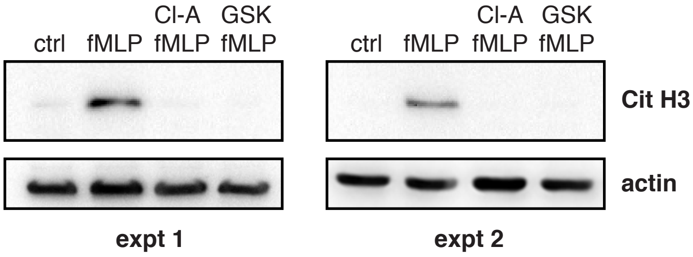

Figure S5. Efficacy of PAD inhibitors in human neutrophils.
Cells were pretreated with 10 µM chloraminidine (“Cl-A”, a general PAD inhibitor), 10 µM GSK484 (a PAD4 inhibitor), or their diluent (DMSO), prior to a further 30-min incubation in the absence (“ctrl”) or presence of 30 nM fMLP. Samples were then processed for immunoblot analysis of citrullinated histone H3. The same samples were also migrated on a parallel gel and immunoblotted for β-actin as a loading control. Two independent experiments are shown.
